# Remnant cholesterol and the risk of carotid plaque in hypertension: results from a community-based screening among old adults in Hangzhou, China

**DOI:** 10.1038/s41598-024-58484-y

**Published:** 2024-04-10

**Authors:** Zhecong Yu, Haifeng Yang, Biqi Shou, Zongxue Cheng, Caixia Jiang, Yang Ye, Jue Xu

**Affiliations:** 1 Institute for Chronic Non-Communicable Disease Control and Prevention, Hangzhou Center for Disease Control and Prevention, Hangzhou, 310000 People’s Republic of China; 2 Fuyang Center for Disease Control and Prevention, Hangzhou, 311400 People’s Republic of China; 3 Tonglu Center for Disease Control and Prevention, Hangzhou, 311400 People’s Republic of China

**Keywords:** Remnant cholesterol, Carotid plaque, Hypertension, Community-based screening, Old adults, Endocrine system and metabolic diseases, Geriatrics, Epidemiology, Biomarkers, Cardiology, Risk factors

## Abstract

Elevated remnant cholesterol (RC) is considered a risk factor for atherosclerotic cardiovascular disease, but the evidence on this association applies to the Chinese population with hypertension is limited. We aimed to explore the association between RC levels and carotid plaque in old adults with hypertension. 8523 hypertensive patients aged ≥ 60 years with serum lipids and carotid ultrasonography data were included in this community-based screening. Fasting RC was calculated as total cholesterol minus high-density lipoprotein cholesterol minus low-density lipoprotein cholesterol (LDLC). The associations of RC levels with carotid plaque risk were evaluated using Logistic regression and restricted cubic spline models. Carotid plaque was screened in 4821 (56.56%) subjects. After multivariable-adjusted, RC was significantly related to carotid plaque [Odd ratio (OR)] = 1.043 per 0.1 mmol/L increase, 95% confidence interval (CI): 1.030–1.056). The highest versus the lowest quartile of RC was 1.928 (1.673–2.223) for carotid plaque. A nonlinear association was found between serum RC levels and the risk of carotid plaque (P for nonlinearity < 0.001). Moreover, an RC > 0.78 mmol/L differentiated patients at a higher risk of carotid plaque compared to those at lower concentrations, regardless of whether LDLC was on target at 2.59 mmol/L. In old adults with hypertension, elevated RC was positively associated with carotid plaque, independent of LDLC and other conventional risk factors.

## Introduction

Carotid plaque formation is a significant risk factor for atherosclerotic cardiovascular disease (ASCVD), which is associated with future risk of stroke, transient ischemic attack, and acute myocardial infarction^1,2^. ~ 30% of Chinese have carotid plaques, and ~ 45% of the old adults are aged ≥ 60 years. The progression of carotid artery atherosclerosis increases more rapidly with age in Chinese than in Europeans^3^. Reducing low-density lipoprotein cholesterol (LDLC) can decrease atherosclerotic risk in clinical practice^4^, but a significant residual cardiovascular risk persists in individuals treated with statins^5,6^. This residual risk may be partly due to the increased concentration of remnant cholesterol (RC)^7–9^. RC is the cholesterol content of triglyceride-rich lipoproteins (TGRL). It comprises very low-density lipoprotein (VLDL) and intermediate-density lipoprotein in the fasting state and chylomicron remnants in the non-fasting state^10^. Like LDLC, RC can be trapped in the intima of the arterial wall, causing arterial intimal cholesterol deposition, vascular inflammation and subsequent plaque formation^11,12^. Several observational population-based studies have shown that elevated RC accelerated common carotid artery intima-media thickness (cIMT), promoted carotid plaque formation, and even increased the risk of atherosclerotic plaque rupture^13–16^, while these studies were mainly conducted in north China and the sample sizes were not large. Notably, an RC increase preceded the development of hypertension^17^, and dyslipidemia combined with hypertension could elevate atherosclerosis risk^18–20^. Limited studies have investigated the relationship between RC and carotid plaque in hypertension. Patients with hypertension are at higher risk of atherosclerosis risk^21^. Accurate identification of high-risk patients in the early stages of atherosclerosis events is necessary to control atherosclerosis progression timely.

Therefore, the present study aimed to evaluate the association between RC and the risk of carotid plaque in hypertension through a community-based screening. Additionally, we investigated whether the risk of carotid plaque associated with RC was independent of LDLC.

## Methods

### Study design and population

We conducted a study collecting individual-level data from a community-based screening of “High-Risk Stroke Population Screening and Intervention”. This screening was supported by the local Centers for Disease Control and Prevention (CDC) and the medical service community between May and July 2020 in Fuyang, Hangzhou. The participants with hypertension and/or diabetes ≥ 60 years old were selected from the service contractor of the family doctor of their community health service centres, and they would also be informed of the details of the screening, including a questionnaire survey, physical examination, and carotid ultrasonography, by their family doctor. Participants meeting the following criteria were excluded from the screening: (1) Mental disorders; (2) Long-term disability; (3) Refuse to attend follow-up. This screening included a sample of 15,561 participants according to the above exclusion criteria. For the present study, of the 11,433 participants with hypertension who were included, 2910 participants with incomplete information on carotid artery imaging, biochemical indexes or conventional risk factors were excluded. Finally, 8523 participants were included in this analysis (Supplementary Fig. 1).

### Data collection

In this study, a trained community health worker collected detailed information on the risk factors of stroke using a standard questionnaire: demographic variables (sex and age), lifestyle (smoking and physical activity), histories of disease (stroke, transient ischemic attacks, and atrial fibrillation/valvular heart disease), and medication information. Smoking was defined as continuous or cumulative smoking for 6 months or more in a lifetime. Regular exercise was defined as exercise ≥ 3 times per week and moderate or above intensity exercise ≥ 30 min per time or engaged in moderate or heavy manual labour. The disease history records required a diagnosis from a middle or above-grade hospital. Height and weight were measured with participants wearing light dressing and without shoes. Body mass index (BMI) was calculated as weight/height^2^ (kg/m^2^). Blood pressure was measured using a standardized automatic electronic sphygmomanometer (HEM-741C; Omron, Tokyo, Japan).

### Laboratory measurements

Blood samples were collected in the morning from each participant's antecubital vein who had fasted for at least 8 h.

Serum creatinine was measured by the alkaline picrate method. The estimated glomerular filtration rate (eGFR) was calculated as follows: eGFR = 175 × Scr^−1.234^ × age^−0.179^(if female, × 0.79), where Scr is serum creatinine concentration (in mg/dL) and age in years^22^. Fasting glucose was measured by glucose-oxidase method, total cholesterol (TC) by esterase oxidase-peroxidase, triglycerides (TG) by glycerol phosphate oxidase-peroxidase, and high-density lipoprotein cholesterol (HDLC) by direct measurement after phosphotungstate precipitation. LDLC was calculated by the Friedewald formula when plasma triglycerides were ≤ 4.0 mmol/L and otherwise measured directly^23^. RC was estimated from a standard lipid profile in the fasting state as TC minus LDLC minus HDLC. The above blood indicators were analyzed using the Roche Cobas automatic biochemical analyzer in an accredited central laboratory in the First People's Hospital of Fuyang (Hangzhou, China), and all laboratory equipment has been calibrated to reduce systematic error effectively.

### Ultrasound examination

Carotid ultrasonography was performed by qualified sonographers who received unified training from the local medical service community, including learning the protocol of screening, unifying the operation procedure of carotid artery ultrasonography, and mastering emergency skills and methods. All sonographers had a professional certificate for ultrasound measurement awarded by the Ministry of Health of China and were blinded to the questionnaire results. High-resolution sonography machines (LOGIQ E9, Chicago, IL, USA) were used for carotid ultrasound with a 10-MHz probe. All participants were studied supine with their heads turned 45 degrees from the site being scanned. The carotid plaques were examined on the left and right sides, with each side measured at three different locations: common carotid artery, carotid bifurcation, and internal carotid artery, and the cIMT was measured. Carotid plaques were defined as focal structures encroaching into the arterial lumen of at least 0.5 mm or 50% of the surrounding cIMT value or demonstrating a thickness > 1.5 mm as measured from the intima-lumen interface to the media-adventitia interface^24^. The ultrasonography results were reviewed by two independent sonographers, and discrepancies were resolved by consensus.

### Statistical analysis

Continuous variables were presented as the means and standard deviation (SD), and categorical variables were expressed as numbers (percentages). Values between groups were compared using variance (ANOVA) or independent t-test and Chi-square test or Fisher’s exact test, as appropriate. Logistic regressions were conducted to assess the association between remnant cholesterol levels and carotid plaque and to calculate the odds ratios (ORs) and 95% confidence intervals (CIs). We constructed 3 models: In model 1, we unadjusted; in model 2, we adjusted for sex and age; in model 3, we adjusted for sex, age, and a propensity score. The propensity score was calculated with a linear regression model entering RC as the dependent variable. The independent variables included smoking, regular exercise, history of stroke, history of transient ischemic attacks (TIA), history of atrial fibrillation /valvular heart disease, family history of stroke, diabetes, BMI, blood glucose, TG, LDLC, HDLC, eGFR, SBP, DBP, antihypertensive medications, and lipid-lowing medications. In these multivariable Logistic models, RC was grouped into quartiles: Q4, > 0.94 mmol/L; Q3, 0.65–0.94 mmol/L; Q2, 0.44–0.65 mmol/L; Q1, ≤ 0.44 mmol/L. RC was entered as a categorical variable with Q1 as the reference group or as a continuous variable per 0.1 mmol/L (3.89 mg/dL) increment. A restricted cubic spline with three knots at the 25th, 50th, and 75th centiles was conducted to flexibly model the association between the RC and carotid plaque risk with adjusted model 3. In addition, we calculated the predicted probabilities of the Logistic model adjusted with/without adjusting for RC, constructed receiver operating characteristic (ROC), and computed the areas under the curves (AUC) to assess the predictive power of each model. Subgroup analyses were also carried out using multivariable Logistic models stratified by traditional risk factors and summarized with forest plots. An interaction term within each subgroup was performed using the likelihood ratio test. For sensitivity analysis, we analyzed the association between RC levels and carotid plaque by excluding participants who were taking lipid-lowering medications. We also employed the E-value as a measure to evaluate the stability of Logistic regression models. This metric aids in assessing whether an observed association may be influenced by an unmeasured underlying factor. Statistical analysis was conducted using SAS 9.4 (SAS Institute Inc., Cary, NC, USA). Two-sided *P*-values < 0.05 were considered statistically significant.

### Ethics approval and consent to participate

The study was conducted in accordance with the Declaration of Helsinki, and approved by the Hangzhou Center for Disease Control and Prevention Research Ethics Committee (Ethics approval number: 2023-5). Informed consent was obtained from all individual participants included in the study.

## Results

### Participants characteristics

The characteristics of study participants were presented in RC quartiles in Table 1. The mean age was 69.91 ± 5.64 years, and 54.99% of participants were female. Participants in the highest quartiles of RC were more likely to be female and had a higher BMI, blood glucose, TC, and TG as compared to the other groups (all *P* < 0.001). The proportions of history of cardiovascular diseases, diabetes, and smoking were more frequent in participants with the lowest quartiles of RC (all *P* < 0.001), while the age of participants was relatively older in the lowest quartiles of RC. The median RC was 0.65 mmol/L (interquartile range, 0.44–0.94 mmol/L). Participants with carotid plaque had a higher level of TC, TG, LDLC, and RC and a lower level of HDLC (Supplemental Table 1). **S**moking, history of atrial fibrillation/valvular heart disease, diabetes, TG, HDLC, LDLC, BMI, SBP, and DBP were independently associated with RC levels (Supplemental Table 2).

**Table 1:** Characteristics of participants by remnant cholesterol quartiles.

Characteristics	Total	Q1	Q2	Q3	Q4	*P*-value	
mmol/L		≤ 0.44	0.44–0.65	0.65–0.94	> 0.94		
N	8523	2160	2133	2117	2113	–	
Demographic							
Age, years	69.91 ± 5.64	70.74 ± 5.80	70.06 ± 5.60	69.79 ± 5.59	69.01 ± 5.41	< 0.001	
Female, n (%)	4687 (54.99)	964 (44.63)	1146 (53.73)	1260 (59.52)	1317 (62.33)	< 0.001	
Lifestyle							
Smoking, n (%)	1428 (16.75)	465 (21.53)	381 (17.86)	318 (15.02)	264 (12.49)		< 0.001
Regular exercise, n (%)	2902 (34.05)	766 (35.46)	715 (33.52)	716 (33.82)	705 (33.36)		0.444
History of cardiovascular diseases							
Stroke, n (%)	577 (6.77)	190 (8.8)	159 (7.45)	128 (6.05)	100 (4.73)	< 0.001	
TIA, n (%)	529 (6.21)	166 (7.69)	130 (6.09)	135 (6.38)	98 (4.64)	< 0.001	
Atrial fibrillation/valvular heart disease, n (%)	1032 (12.11)	322 (14.91)	241 (11.3)	254 (12.0)	215 (10.18)	< 0.001	
Diabetes, n (%)	1767 (20.73)	479 (22.18)	402 (18.85)	427 (20.17)	459 (21.72)	0.029	
Family history of stroke, n (%)	994 (11.66)	239 (11.06)	269 (12.61)	251 (11.86)	235 (11.12)	0.349	
Laboratory values							
Blood glucose, mmol/L	5.98 ± 1.50	5.91 ± 1.46	5.93 ± 1.46	5.94 ± 1.41	6.15 ± 1.64	< 0.001	
TC, mmol/L	4.78 ± 1.09	4.15 ± 0.91	4.58 ± 0.91	4.96 ± 0.91	5.45 ± 1.16	< 0.001	
TG, mmol/L	1.84 ± 1.38	1.17 ± 0.79	1.46 ± 0.66	1.84 ± 0.86	2.90 ± 2.02	< 0.001	
HDLC, mmol/L	1.40 ± 0.36	1.49 ± 0.37	1.42 ± 0.35	1.40 ± 0.34	1.29 ± 0.33	< 0.001	
LDLC, mmol/L	2.63 ± 0.81	2.37 ± 0.78	2.61 ± 0.79	2.78 ± 0.80	2.75 ± 0.81	< 0.001	
eGFR, mL/min per 1.73m^2^	63.22 ± 40.20	64.75 ± 28.88	63.40 ± 39.25	62.35 ± 44.46	62.36 ± 46.13	0.162	
Physical values							
BMI, kg/m2	19.45 ± 2.71	19.22 ± 2.73	19.43 ± 2.76	19.50 ± 2.63	19.67 ± 2.71	< 0.001	
SBP, mm Hg	132.79 ± 14.54	133.10 ± 14.57	133.31 ± 15.11	132.81 ± 14.69	131.94 ± 13.72	0.012	
DBP, mm Hg	77.01 ± 8.29	76.60 ± 8.32	77.25 ± 8.40	77.06 ± 8.32	77.12 ± 8.11	0.060	
Antihypertensive medications							
ACEI, n (%)	8409 (98.66)	2137 (98.94)	2105 (98.69)	2087 (98.58)	2080 (98.44)	0.545	
ARB, n (%)	4185 (49.1)	1051 (48.66)	1021 (47.87)	1053 (49.74)	1060 (50.17)	0.428	
β-blockers, n (%)	421 (4.94)	122 (5.65)	110 (5.16)	91 (4.30)	98 (4.64)	0.188	
CCB, n (%)	4699 (55.13)	1182 (54.72)	1217 (57.06)	1176 (55.55)	1124 (53.19)	0.082	
Diuretics, n (%)	1891 (22.19)	456 (21.11)	476 (22.32)	483 (22.82)	476 (22.53)	0.553	
Lipid-lowering medications, n (%)	49 (0.57)	18 (0.21)	15 (0.18)	8 (0.09)	8 (0.09)	0.112	

Data were shown as mean ± SD for continuous variables and n (%) for categorical variables. *BMI* body mass index; *TIA* transient ischemic attacks; *SBP* systolic blood pressure; *DBP* diastolic blood pressure; *TC* total cholesterol; *TG* triglycerides; *HDLC* high-density lipoprotein cholesterol; *LDLC* low-density lipoprotein cholesterol; *eGFR* estimated glomerular filtration rate; *ACEI* angiotensin-converting enzyme inhibitor; *ARB* angiotensin receptor blocker; *CCB* calcium channel blocker.

### RC and carotid plaque risk

**Figure 1:**
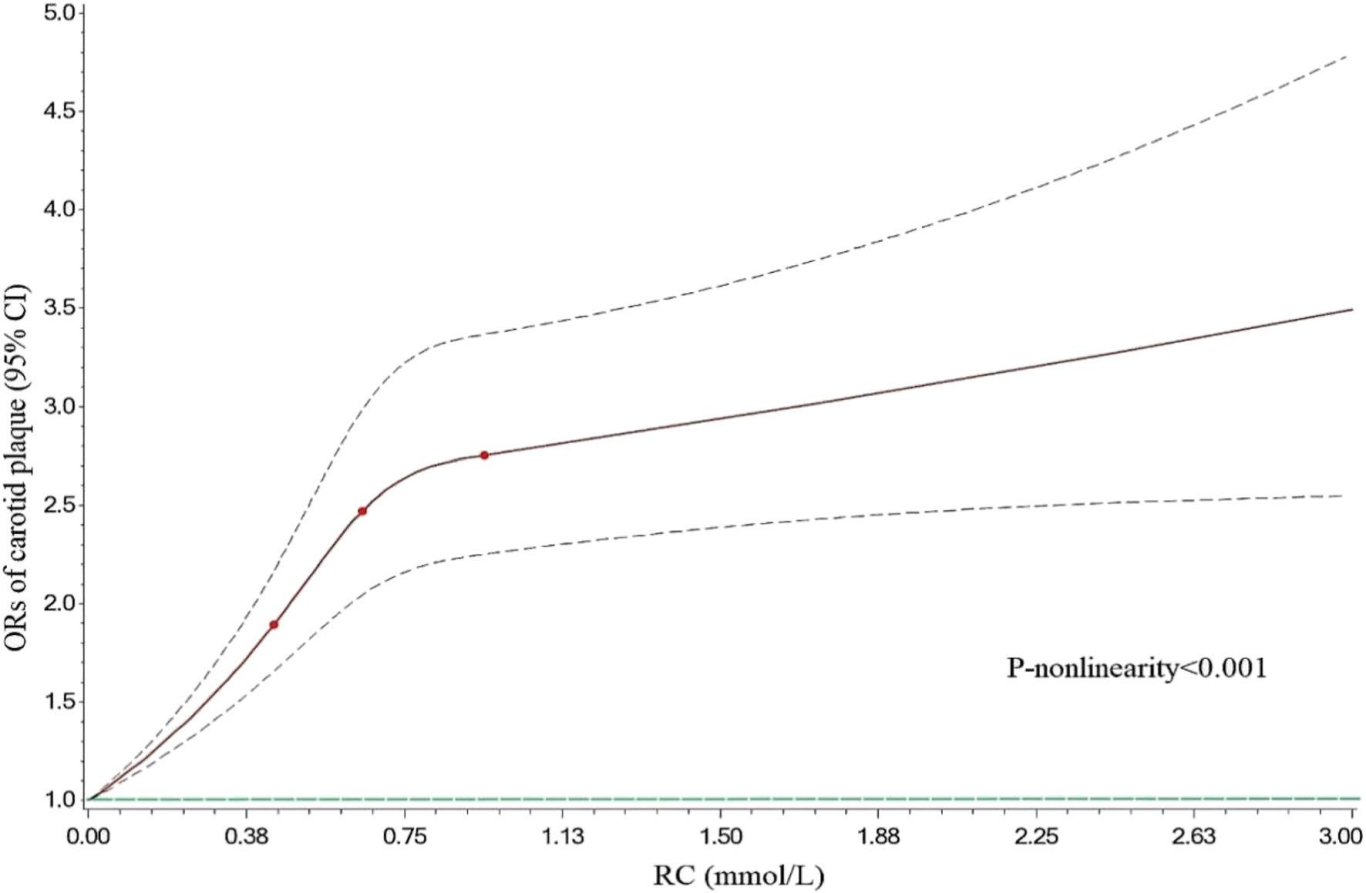
Association of remnant cholesterol with carotid plaque. Red lines indicate hazard ratios, green line indicates the reference line (The minimum of RC as a reference point), and dashed lines indicate 95% CI from restricted cubic spline regression. ORs were calculated using Logistic regression analysis after adjustments for sex, age and a propensity score. RC remnant cholesterol; OR odds ratio; CI confidence interval.

**Figure 2:**
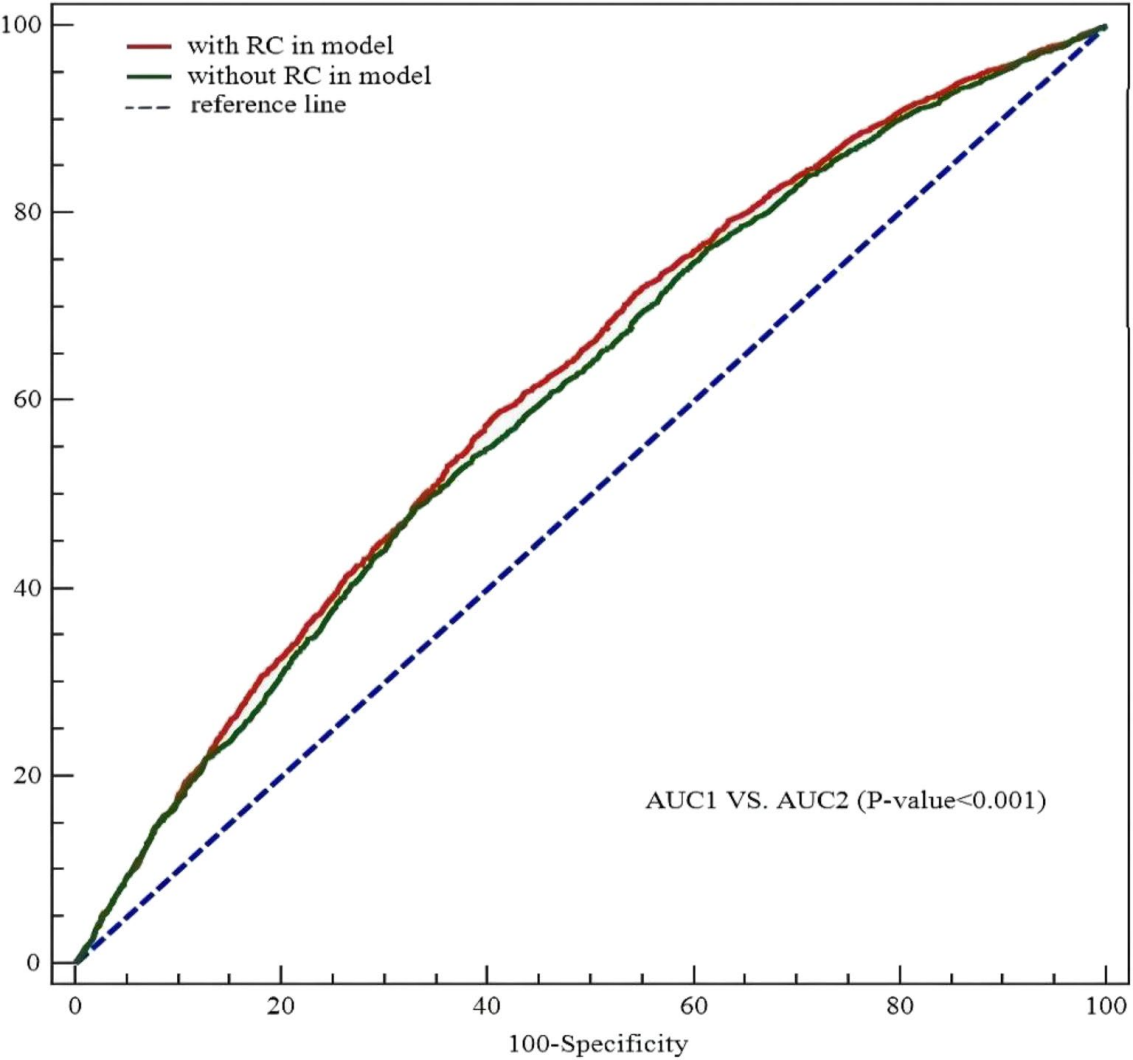
Receiving operating characteristics curves for carotid plaque from Logistic regression models with and without remnant cholesterol in patients with hypertension. AUCs were performed containing: (1) sex, age and a propensity score, and (2) RC added. A comparison between the AUCs is noted in the bottom panel. RC remnant cholesterol; AUC areas under the curves.

**Figure 3:**
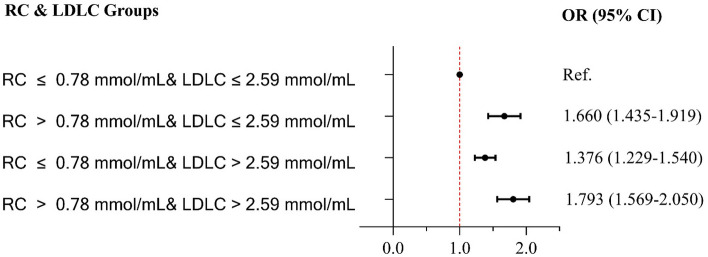
Risk of carotid plaque based on categories of remnant cholesterol and LDLC. Data were adjusted for age, sex, smoking, regular exercise, history of stroke, history of TIA, history of atrial fibrillation/valvular heart disease, family history of stroke, diabetes, blood glucose, BMI, TG, HDLC, eGFR, SBP, DBP, antihypertensive medications, and lipid-lowing medications. OR odds ratio; CI confidence interval. Other abbreviations as in Table 1.

Multivariate Logistic regression analysis revealed that (Table 2), in model 1 (crude model), compared with Q1, participants with higher RC levels were significantly associated with an increased risk of carotid plaque (Q2: OR 1.555, 95% CI 1.378–1.754; Q3: OR 1.838, 95% CI 1.628–2.075; and Q4: OR 2.157, 95% CI 1.908–2.439). In the fully adjusted model (model 3), RC remained a significant risk factor for carotid plaque (Q2 vs. Q1: OR 1.489, 95% CI 1.316–1.683; Q3 vs. Q1: OR 1.721, 95% CI 1.516–1.953; and Q4 vs. Q1: OR 1.928, 95% CI 1.673–2.223; per 0.1 mmol/L increment: OR 1.043, 95%CI 1.030–1.056). In Fig. 1, the restricted cubic splines showed that higher levels of RC were associated with a progressively increased risk of carotid plaque (P-nonlinearity < 0.001). There was a substantial increase in the associated risk until around 0.75 mmol/L (29.18 mg/dL). The ROC analysis based on Logistic models without/with RC was presented in Fig. 2. The first model had an AUC of 0.605 (95% CI: 0.594–0.615). The second model included RC enabled a more accurate prediction of carotid plaque (AUC 0.617, 95% CI 0.606–0.627) and showed a significant model improvement (ΔAUC 0.012, 95% CI 0.007–0.017, *P* < 0.001 by DeLong’s test). Based on the combinations of each RC and LDLC cutoff value^9^, participants were categorized into four groups (Fig. 3). Compared to lower concentrations of RC within the same LDLC levels, even in participants with controlled LDLC, high RC was associated with a 66.0% higher risk of carotid plaque in RC&LDLC (> 0.78 & ≤ 2.59 mmol/L) group (OR 1.660, 95% CI 1.435–1.919).

**Table 2:** Association between remnant cholesterol and risk of carotid plaque.

	Model 1	Model 2	Model 3
	OR (95% CI)	OR (95% CI)	OR (95% CI)
Remnant cholesterol (per 0.1 mmol/L increase)	1.052 (1.042–1.062)	1.042 (1.032–1.052)	1.043 (1.030–1.056)
Remnant cholesterol (quartiles)			
Q2 vs. Q1	1.555 (1.378–1.754)	1.489 (1.318–1.683)	1.489 (1.316–1.683)
Q3 vs. Q1	1.838 (1.628–2.075)	1.722 (1.522–1.949)	1.721 (1.516–1.953)
Q4 vs. Q1	2.157 (1.908–2.439)	1.934 (1.706–2.192)	1.928 (1.673–2.223)

Model 1 adjusted none; Model 2 adjusted for sex and age; Model 3 adjusted for sex, age and a propensity score. *OR* odds ratio; *CI* confidence interval.

### Subgroup analysis

**Figure 4:**
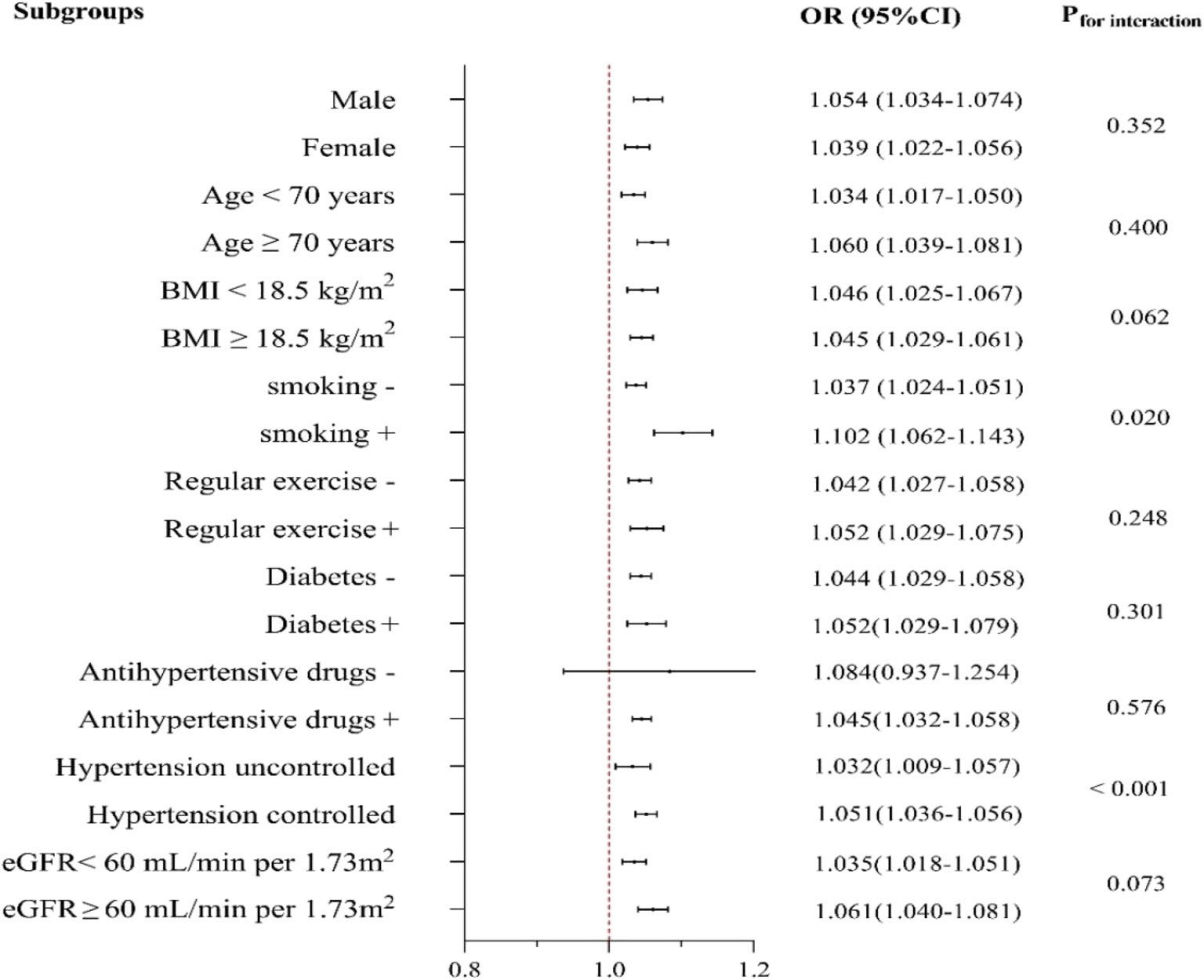
Risk of carotid plaque for the highest vs. lowest quartile of remnant cholesterol in various subgroups adjusted for sex, age, smoking, regular exercise, history of stroke, history of TIA, history of atrial fibrillation/valvular heart disease, family history of stroke, diabetes, BMI, blood glucose, SBP, DBP, TG, LDLC, HDLC, eGFR, antihypertensive medications, and lipid-lowering medications other than variables for stratification. ORs for per 0.1 mmol/L increased in RC. *P*-value for interaction was calculated. Hypertension uncontrolled was defined as SBP ≥ 140 and/ or DBP ≥ 90 mmHg in this screening. OR odds ratio; CI confidence interval. Other abbreviations as in Table 1.

Sex, age, BMI, smoking, regular exercise, diabetes, antihypertensive drug, hypertension control and eGFR levels were stratified for further analysis of the association between RC and carotid plaque (Fig. 4). Positive associations were found in various subgroups, except for associations stratified by antihypertensive medications. The higher risk of carotid plaque with RC levels persisted notably, regardless of sex, age, BMI, regular exercise, diabetes and eGFR levels. The association between RC level and carotid plaque risk was stronger in smoking and in participants with controlled hypertension.

### Sensitivity analysis

When excluding participants who were taking lipid-lowering medications, the results did not change substantially. In model 3, RC was positively correlated with the carotid plaque risk (per 0.1 mmol/L increment, OR 1.043, 95% CI 1.030–1.055). Compared with Q1, participants with higher RC levels were still significantly associated with an increased risk of carotid plaque (Q2: OR 1.487, 95% CI 1.315–1.681; Q3: OR 1.717, 95% CI 1.512–1.949; and Q4: OR 1.919, 95% CI 1.665–2.212) (Supplemental Table 3). We also calculated an E-value calculation to assess the potential impact of unaccounted confounders. An E-value of 1.17, with a lower bound of the 95% CI at 1.14. These confounding factors would need to have an OR of at least 1.17 to account for the observed association (Supplemental Fig. 2).

## Discussion

Our results demonstrate that in older adults with hypertension, the association between RC and risk of carotid plaque was nonlinear. Higher RC levels were associated with an increased risk of carotid plaque, even in participants at the target range of LDLC levels. Adding the RC to a traditional risk model had an incremental effect on the discriminative ability of carotid plaque.

The association between high RC and ASCVD has been explored in numerous studies^25^, which indicated that elevated RC levels could increase the risk of ASCVD as well as coronary heart disease and stroke. The evidence for the risk of carotid plaque, as a surrogate marker of ASCVD risk^26^, with RC would be valuable. Cross-sectional studies showed that elevated fasting RC concentrations were positively associated with mean cIMT and maximum cIMT in 1496 patients with ischemic stroke^13^, and a high RC level indicated a higher risk of intra- and extra-cranial atherosclerosis in 3665 community residents^16^. Even among 767 youth, elevated serum RC levels were associated with increased cIMT^27^. Consistently, coronary artery disease patients with high RC are more likely to have atherosclerotic plaque and plaque rupture^15,28^. The above findings were in line with ours, whereas their sample size was not large, or the age distribution of the study population was different. The present study observed a nonlinear positive association between RC levels and carotid plaque risk in hypertension. Maintaining RC levels as low as possible was recommended to reduce the risk of carotid plaque. The effect of hypertension on RC-carotid plaque links was inconclusive. History of hypertension did not modify the relationship between RC and vascular disease^13,29^, while high RC levels could potentiate hypertension development and increase the risk of first-ever stroke, partially through the pathway of hypertension^17,30^. The increased carotid plaque risk associated with a high RC level in hypertension is due, in part, to BP-related atherogenic dyslipidemia. Hypertension is closely related to dyslipidemia, which is characterized by the increase of plasma triglyceride, VLDL, and atherogenic LDLC particles and the decrease of HLDC^31^. Interestingly, a study of 45 severe carotid artery stenosis patients found that cholesterol in the dense LDL and in the triglyceride-rich lipoprotein remnants, not LDLC, HDLC or TG levels, affected carotid plaque and carotid plaque inflammatory cellular composition^32^. On the one hand, TG is unlikely to be an independent risk factor for vascular disease^33^. Although according to the used Friedewald equation in the present study, RC equals TG divided by 5, TG could not directly cause atherosclerotic plaques. TG-rich chylomicrons are too large to enter the arterial wall and are phagocytosed by vascular macrophages^34^. Nordestgaard et al. reported that the harmful component in TRLs was cholesterol, not TG itself^10^. On the other hand, studies on the effect of elevated HDLC levels on vascular disease risk were inconsistent. Valentina et al. reported a nonlinear U-shaped association between HDLC levels and CVD risk in 6523 males with hypertension but not females^35^. Lipoproteomics revealed that medium and large HDL subclasses levels were negatively associated with higher cIMT, whereas very large HDL were positively associated with carotid plaque^36^. The increase in HDLC levels may fail to reduce vascular disease risk^37^. These findings supported that TG may just be a marker of elevated RC. Thus, the assessment of RC, an independent residual risk factor for hypertensive patients, could be more meaningful than TG or HDLC to provide additional information on the primary prevention of ASCVD.

In the present study, ROC analysis revealed that adding RC into a model resulted in a significant improvement in its discriminant ability of risk of carotid plaque in hypertensive patients. Our study implied that the assessment of RC may help improve the prognostic risk of future carotid plaque information in old adults with hypertension. In addition, we pointed out that the median RC was 0.65 mmol/l (interquartile range, 0.44–0.94 mmol/l), which was similar to that of the stroke patients but higher than that of the general population^13,14^. Ageing and hypertension may partly explain the relatively high RC concentrations. Future natural population cohort studies are needed to estimate the concentration distribution of RC to obtain a normal or low-risk physiological concentration range.

In this study, we also found that individuals with high RC concentration (> 0.78 mmol/L) had a higher risk of carotid plaque, regardless of whether LDLC concentration was or was not at optimal values (≤ 2.59 mmol/L). This finding was consistent with others from previous studies which were conducted in specific ethnic and disease groups^25^. Our conclusions extended these prior findings and implicated that for patients at the target LDLC levels, treatments to control RC to reduce the residual risk of ASCVD were more beneficial. In the subgroup analysis, we did not find an association between RC and carotid plaque among participants who did not take antihypertensive medications. We assumed the following possible explanations: First, the sample may be insufficient, with only 1.33% (114/8523). An analysis of this factor stratification may be underpowered. Additionally, with regards to hypertension control, given the specificity of the effects of antihypertensive medications on lipid metabolism^38,39^, the dosage and frequency should also be collected in future studies to evaluate the lipid metabolism in patients with hypertension comprehensively.

### Potential mechanistic links

Previous observational and genetic epidemiological data have indicated a causal role of cholesterol content within TGRL and/or RC in the development of ASCVD. The strong effect of RC on ASCVD can be explained by the following mechanisms. Remnant lipoprotein cholesterol penetrates the endothelial layer and traps into the arterial intima. However, unlike LDLC, it can be absorbed directly by macrophages, which contributes to the formation of foam cells and atherosclerotic plaques^40^. RC is larger than LDLC and carries up to 40 times more cholesterol per particle, which may make RC more likely to cause arteriosclerosis than LDLC^41^. Elevated RC is related to low-grade inflammation^11^, causing arterial wall inflammation and a multilevel cellular immune response^12^. It is also related to the content of macrophages in the carotid artery, which is a marker of plaque instability^32^. RC is regarded as an indicator of endothelial vasomotor dysfunction and upregulates the expression of proatherothrombogenic molecules mediated by redox-sensitive mechanisms^42^. In summary, the combination of arterial intima accumulation, inflammation and endothelial vasomotor dysfunction could partially explain the increased risk of arterial plaque formation.

### Merits and limitations

The main strength of this study was that we reported novel associations between RC and carotid plaque in hypertension in Chinese old adults. Our findings provided community-based evidence to support the hypothesis that RC was a risk factor for ASCVD and served as an explanation for the mechanism of RC-CVD links. Besides, RC could be obtained from the standard lipid profile without additional cost. This measurement was beneficial to be widely used in clinical practice to identify high-risk patients early.

Some limitations should be considered in the light of the present results. Firstly, the present study population was restricted to an old adults hypertensive sample in East China, which induced some selection bias and limited the generalizability of the present results. Secondly, given the nature of the cross-sectional study, the dynamic change effects of RC levels on carotid plaque formation were unable to be assess. A prospective cohort study was needed to clarify a cause-effect. Thirdly, we use fasting lipid samples to calculate RC. Thus, in the non-fasting state, it remains plausible that chylomicron remnants partially contribute to carotid plaque. However, RC was slightly but not clinically significant after a habitual meal. In this study, the strength of association between RC and carotid plaque may be underestimated by the use of fasting samples, but the association was still significant after the multivariate adjustment. Similar studies have also used fasting samples to calculate RC^25^, as in our study. Fourthly, carotid artery imaging was operated by a single sonographer, and we could not evaluate inter-rater variability for the measurements and the residual bias from diagnostic suspicion. Finally, some deficiencies may exist in the information on lifestyle factors, hypertension duration, and medication adherence. These could affect lipid metabolism and introduce residual bias.

## Conclusions

In conclusion, elevated levels of RC were associated with carotid plaque in patients with hypertension independently of traditional cardiovascular risk factors. Our findings strengthened the evidence for the necessity of monitoring RC for vascular health in hypertension. RC may serve as a potential risk factor in community prevention and clinical intervention, even in people at optimal LDLC levels.

### Supplementary Information

Supplementary Information.

## Data Availability

The data presented in this study are available on request from the corresponding authors. The data are not publicly available due to ethical, legal, and privacy issues.
