# Supplementary material for: Remnant cholesterol and the risk of carotid plaque in hypertension: results from a community-based screening among old adults in Hangzhou, China
Source: Sci Rep. 2024 Apr 10;14:8407. doi: 10.1038/s41598-024-58484-y (PMC11006856; doi:10.1038/s41598-024-58484-y)
Supplement: Supplementary file 1 — Supplementary Information. [file 41598_2024_58484_MOESM1_ESM.docx]

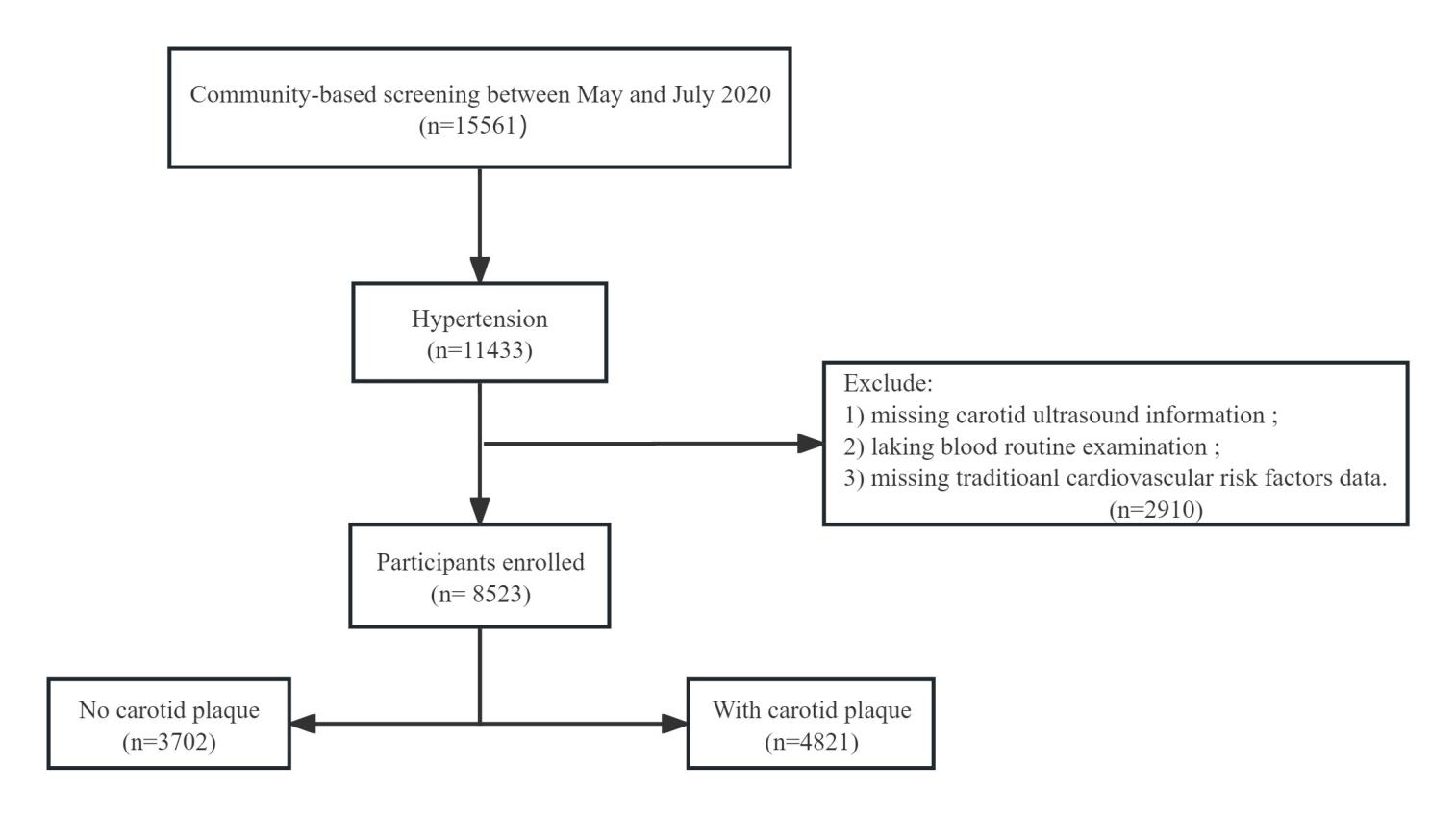


**Supplemental Figure 1.** Flowchart of the study.

| **Supplemental Table 1** Lipid values by carotid plaque. | | | |
| --- | --- | --- | --- |
|  | Without carotid plaque | With carotid plaque | P-value |
| TC, mmol/L | 4.67±1.09 | 4.87±1.08 | < 0.001 |
| TG, mmol/L | 1.77±1.29 | 1.89±1.44 | < 0.001 |
| HDLC, mmol/L | 1.42±0.36 | 1.39±0.35 | <0.001 |
| LDLC, mmol/L | 2.56±0.85 | 2.68±0.78 | < 0.001 |
| RC, mmol/L | 0.69±0.50 | 0.81±0.60 | <0.001 |
| RC & LDLC groups |  |  | <0.001 |
| RC ≤ 0.78 mmol/L & LDLC ≤ 2.59 mmol/L | 1518 (41.00) | 1395 (28.94) |  |
| RC > 0.78 mmol/L & LDLC ≤ 2.59 mmol/L | 498 (13.45) | 857 (17.78) |  |
| RC ≤ 0.78 mmol/L & LDLC > 2.59 mmol/L | 1062 (28.69) | 1433 (29.72) |  |
| RC > 0.78 mmol/L & LDLC > 2.59 mmol/L | 624 (16.86) | 1136 (23.56) |  |
| Data were shown as mean ± SD or n (%). Abbreviations were same as in Table1. | | | |

| **Supplemental Table 2** Associated factors with remnant cholesterol form multivariable linear regression model | | |
| --- | --- | --- |
| Variables | β coefficient | P-value |
| Smoking | -0.512 | <0.001 |
| Regular exercise | -0.123 | 0.261 |
| stroke | -0.243 | 0.259 |
| TIA | -0.247 | 0.269 |
| Atrial fibrillation /valvular heart disease | -0.374 | 0.020 |
| Diabetes | -0.329 | 0.031 |
| Family history of stroke | -0.105 | 0.515 |
| Blood glucose | -0.032 | 0.443 |
| Triglycerides | 2.006 | <0.001 |
| HDLC | -0.992 | <0.001 |
| LDLC | 0.402 | <0.001 |
| eGFR | 0.000 | 0.803 |
| BMI | -0.047 | 0.019 |
| SBP | -0.017 | <0.001 |
| DBP | 0.015 | 0.023 |
| Antihypertensive medications | -0.160 | 0.722 |
| Lipid-lowering medications | -0.812 | 0.236 |
| β coefficients and p values estimated with linear regression model adjusted for all other characteristics. Abbreviations were same as in Table1. | | |

| **Supplemental Table 3** Remnant cholesterol and carotid plaque in sensitivity analysis among participants without take any lipid-lowering medications (n=8474) | | | | | |
| --- | --- | --- | --- | --- | --- |
|  | Model 1 |  | Model 2 |  | Model 3 |
|  | OR (95% CI) |  | OR (95% CI) |  | OR (95% CI) |
| Remnant cholesterol (per 0.1mmol/L increase) | 1.052 (1.052–1.062) |  | 1.042 (1.032–1.052) |  | 1.043 (1.030–1.055) |
| Remnant cholesterol (quartiles) |  |  |  |  |  |
| Q2 vs. Q1 | 1.555 (1.378–1.754) |  | 1.489 (1.318–1.683) |  | 1.487 (1.315–1.681) |
| Q3 vs. Q1 | 1.838 (1.628–2.075) |  | 1.722 (1.522–1.949) |  | 1.717 (1.512–1.949) |
| Q4 vs. Q1 | 2.157 (1.908–2.439) |  | 1.934 (1.706–2.192) |  | 1.919 (1.665–2.212) |
| Model 1 adjusted none; Model 2 adjusted for sex and age; Model 3 adjusted for sex, age and a propensity score. OR odds ratio; CI, confidence interval. | | | | | |

**
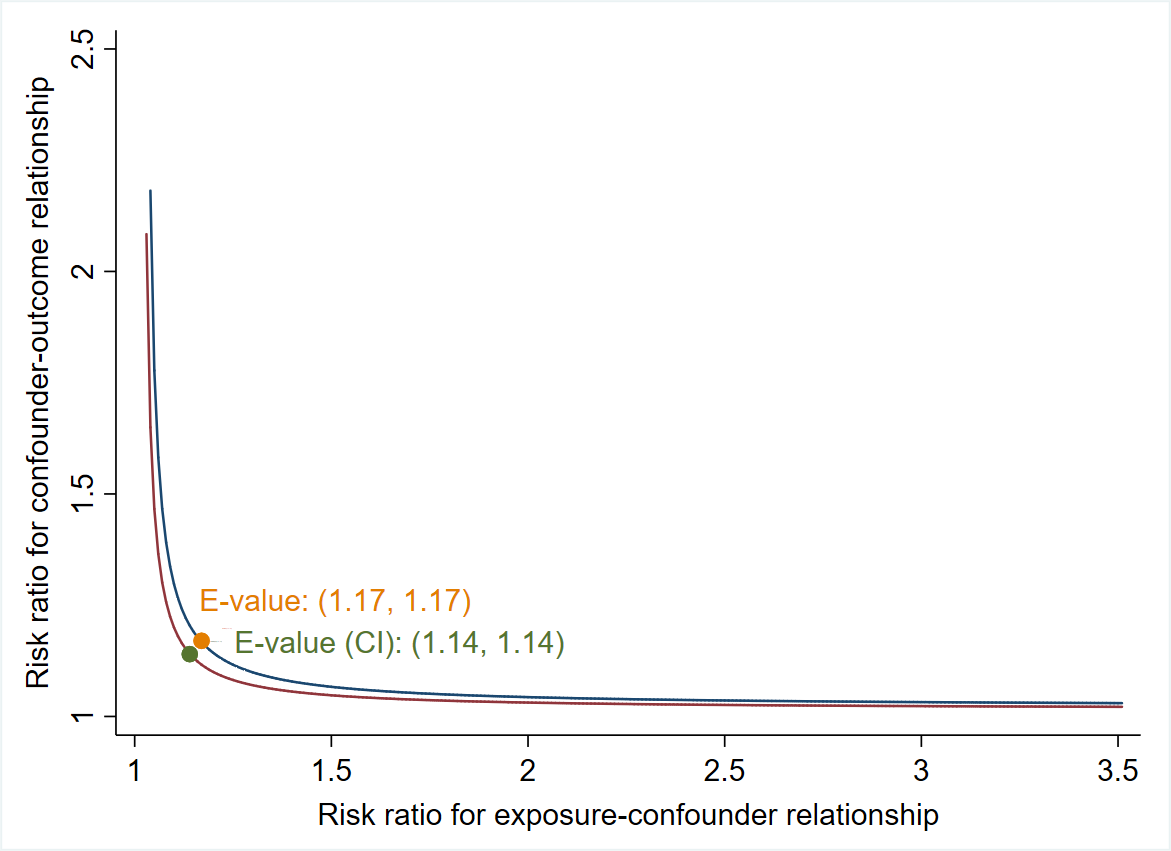
**

**Supplemental Figure 2.** Curves of the sensitivity analysis for unobserved confounders with E-value. Curve depicting the range of joint relationships (RC per 0.1mmol/L increase-confounder and confounder-carotid plaque) that may explain away the estimated effect and its confidence interval for the multivariable logistic regression model to predict carotid plaque. A larger E-value indicates greater robustness of the research conclusion and makes it more difficult for confounding factors to explain or overturn the results.
